# Dietary Zinc Supplementation in Steers Modulates Labile Zinc Concentration and Zinc Transporter Gene Expression in Circulating Immune Cells

**DOI:** 10.1007/s12011-024-04123-6

**Published:** 2024-03-05

**Authors:** Carlos E. Franco, Emma L. Rients, Fabian E. Diaz, Stephanie L. Hansen, Jodi L. McGill

**Affiliations:** 1 Department of Veterinary Microbiology and Preventive Medicine, Iowa State University, 1907 ISU C-Drive, Ames, IA USA; 2 Department of Animal Science, Iowa State University, Ames, IA USA

**Keywords:** Zinc, Zinc transporters, Immune cells, Labile zinc, Feedlot cattle

## Abstract

Zinc (Zn) is critical for immune function, and marginal Zn deficiency in calves can lead to suboptimal growth and increased disease susceptibility. However, in contrast to other trace minerals such as copper, tissue concentrations of Zn do not change readily in conditions of supplementation or marginal deficiency. Therefore, the evaluation of Zn status remains challenging. Zinc transporters are essential for maintaining intracellular Zn homeostasis, and their expression may indicate changes in Zn status in the animal. Here, we investigated the effects of dietary Zn supplementation on labile Zn concentration and Zn transporter gene expression in circulating immune cells isolated from feedlot steers. Eighteen Angus crossbred steers (261 ± 14 kg) were blocked by body weight and randomly assigned to two dietary treatments: a control diet (58 mg Zn/kg DM, no supplemental Zn) or control plus 150 mg Zn/kg DM (HiZn; 207 mg Zn/kg DM total). After 33 days, Zn supplementation increased labile Zn concentrations (as FluoZin-3 fluorescence) in monocytes, granulocytes, and CD4 T cells (*P* < 0.05) but had the opposite effect on CD8 and γδ T cells (*P* < 0.05). Zn transporter gene expression was analyzed on purified immune cell populations collected on days 27 or 28. ZIP11 and ZnT1 gene expression was lower (*P* < 0.05) in CD4 T cells from HiZn compared to controls. Expression of ZIP6 in CD8 T cells (*P* = 0.02) and ZnT7 in B cells (*P* = 0.01) was upregulated in HiZn, while ZnT9 tended (*P* = 0.06) to increase in B cells from HiZn. These results suggest dietary Zn concentration affects both circulating immune cell Zn concentrations and Zn transporter gene expression in healthy steers.

**Supplementary Information:**

The online version contains supplementary material available at 10.1007/s12011-024-04123-6.

## Introduction

Zinc (Zn) is a transition metal abundant in mammalian bodies that plays a crucial role in many cellular mechanisms and physiological processes. It supports innate and adaptive immune function, acts as a structural component in gene expression and signal transduction, and is a cofactor for over 300 enzymes [1–4]. The transition metal Zn can modulate resistance to infection by regulating cell proliferation and differentiation and controlling critical genes and pathways during an immune response [5, 6]. Thus, understanding the underlying mechanism for Zn homeostasis is critical for improving livestock health.

Cattle rely on Zn for optimal growth, immune function, and overall health. Clinical Zn deficiency is rare in cattle, but many animals experience marginal Zn deficiency which can lead to suboptimal growth and increased disease susceptibility through immune dysfunction [7–10]. Most Zn deficiencies can be corrected with dietary Zn supplementation, such as Zn sulfate [11, 12]; current recommendations of Zn for cattle are 30 mg Zn/kg DM [13]. However, accurately determining an animal’s Zn status during marginal Zn deficiency remains challenging. Plasma Zn concentration and liver biopsies are commonly used in humans and cattle to identify severely Zn deficient populations, yet plasma and liver Zn remain insensitive to marginal Zn changes and are affected by other factors such as stress, infection, and other metabolic conditions unrelated to Zn status [14, 15]. Given the crucial role of Zn in immune function, alternative biomarkers are needed.

Two approaches recently evaluated in rodent models and humans are promising alternatives for evaluating Zn status. The first involves FluoZin-3, a fluorescent probe that detects labile Zn, to evaluate changes in Zn concentrations in immune cells via flow cytometry [16], which may provide a more accurate reflection of Zn status than plasma Zn [17–19]. Hamon et al*.* demonstrated that macrophages from the lungs of patients with chronic obstructive pulmonary disease had less intracellular labile Zn compared to those from healthy patients, while plasma Zn did not differ [18]. Reduced concentrations of macrophage labile Zn were correlated with impaired efferocytosis, a critical function for maintaining lung homeostasis [18].

Intracellular Zn homeostasis is regulated by 14 members of the Zrt/Irt-like protein (ZIP) family, 10 members of the Zn transporter protein (ZnT) family, and two Zn-sequestering proteins called metallothioneins (MT) [20–22]. ZIP proteins function to increase cytosolic Zn, and their dysregulation has been linked to various diseases. ZnTs, on the other hand, are responsible for decreasing cytosolic Zn, and their expression levels change depending on Zn status.

The second approach for measuring Zn status is based on the expression of specific Zn transporters in immune cells from blood, which has been shown to fluctuate in response to Zn supplementation or deficiency [23–25]. Chu et al*.* compared the expression of all known ZnTs, ZIPs, and MTs in 39 adults receiving 22 mg/day Zn supplementation and determined that the expression of many Zn transporters changed, with both MT2A and ZIP1 being significant predictors of dietary Zn status [26]. Hennigar et al*.* measured MT, ZnT1, ZIP8, and ZIP11 expression in PBMC from 54 healthy adults and observed that PBMC MT2A expression positively correlated with total Zn intake, while plasma Zn did not [24].

The aim of the study was to determine how dietary Zn supplementation changed labile Zn concentrations and the expression of Zn transporters in circulating immune cells isolated from feedlot steers, with the long-term objective of identifying if these two approaches are potential predictors of Zn status in cattle.

## Materials and Methods

### Animals and Sample Collection

Animals used for this study were part of an overall larger trial described by Rients et al. (manuscript in preparation). Briefly, 72 Angus-crossbred steers (261 ± 14 kg) were stratified by initial body weights (BW) into GrowSafe-equipped pens (*n* = 6 steers/pen). GrowSafe bunks allow for individual feed disappearance to be calculated; thus, the steer was the experimental unit. Dietary treatments were randomly assigned to pens. On day 0, steers were administered a Component E-S with Tylan implant (Elanco Animal Health, Greenfield, IN), and dietary treatments started. Dietary treatments consisted of control with no supplemental Zn and 150 mg of supplemental Zn/kg DM (HiZn). The analyzed Zn concentration for control was 58 mg Zn/kg DM and HiZn was 207 mg Zn/kg DM. Supplemental Zn was added as Zn sulfate (ZnSO_4_), and the diet consisted of Sweetbran, a branded wet corn gluten feed (40%, Cargill), corn silage (40%), dried distillers grains (18%), and vitamins and minerals (1.94%, recommendations based on NASEM 2016). Full diet content is available in Rients et al. (manuscript in preparation). The dietary treatments were delivered as part of the total mixed ration using dried distiller grains as a carrier for treatments and other micronutrients. Liver samples were collected on days − 7, 14, and 58 using methods described by Engle and Spears (2000). Liver was stored at – 20 °C prior to trace mineral analysis. On days − 1, 13, 28, and 57, blood was collected from all steers (BD vacutainer blood collection tube for trace element testing, royal blue top, containing K_2_EDTA), centrifuged at 1000 × *g* for 20 min, aliquoted, and stored at – 20 °C for further analysis. On days 27 and 28, heparinized whole blood was collected from control (*n* = 3 per day) and HiZn (*n* = 3 per day) steers (BD vacutainer heparin tubes, green top), for a total of 6 per treatment. Cells were collected for cell sorting and Zn transporter gene expression analysis. In addition to the initial 12 steers, six more were selected (*n* = 9 steers/treatment) and heparinized whole blood was collected for FluoZin-3 flow analysis on day 33 (BD vacutainer heparin tubes, green top). All animal procedures were approved by the Iowa State University Institutional Animal Care and Use Committee (IACUC-21–150).

### Trace Mineral Concentrations

Plasma and liver samples were analyzed for trace minerals using inductively coupled plasma– optical emissions spectroscopy (ICP-OES; Optima 7000; PerkinElmer, Waltham, MA) using methods described in Pogge and Hansen (2013). Quality control samples (serum UTAK, Valencia, CA; bovine liver from National Institutes of Standards and Technology, Gaithersburg, MD) were included in all runs to verify instrument accuracy.

### FluoZin-3 Assay and Flow Cytometry

**Fig. 1:**
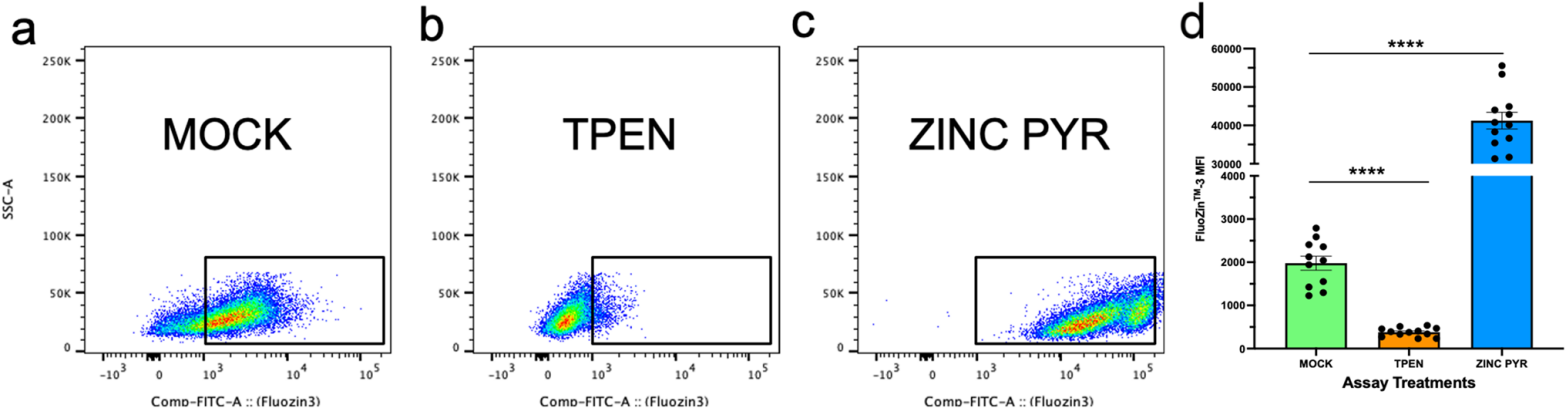
FluoZin™-3 assay validation. Peripheral blood isolated from donor steers were plated (1 × 10^6^) and loaded with FluoZin-3 and respective antibodies as described above, and incubations were carried out at 37 ℃. To determine if changes in labile Zn concentration is reflected by changes in FluoZin-3 fluorescence, cells were incubated for 30 min in three different conditions: **a** in PBS to mimic resting immune cells, **b** in 50 µM of the Zn chelator TPEN to mimic a Zn-depleted/deficiency environment, or **c** in 100 µM of Zn pyrithione to mimic a Zn supplemented environment. Results are shown in flow plots (a) and (b) to show changes in FluoZin-3 fluorescent or in bar graphs (**d**) for a simpler representation. Data represent mean ± SEM. Data were analyzed using a one-way ANOVA with a multiple comparison test

To validate the FluoZin-3 assay, cells isolated from donor steers were plated (1 × 10^6^) and labeled with FluoZin-3 dye and respective antibodies for 30 min, and then incubated with either PBS or with 50 µM of *N*,*N*,*N*′,*N*′-*Tetrakis*(2-pyridylmethyl)ethylenediamine (TPEN, a membrane-permeable heavy metal chelator with high affinity for Zn) to create Zn-depleted cells or 100 µM of Zn chloride with sodium pyrithione to create Zn-supplemented cells. Changes in labile Zn concentrations were then evaluated based on FluoZin-3 fluorescence using flow cytometry, as shown in Fig. 1. The FluoZin-3 assay was performed as described by Haase et al., with minor modifications [17]. Cells from lysed whole blood were loaded with FluoZin-3 AM ester (5 µM) and labeled with designated primary cell surface antibodies: anti-bovine CD14 monoclonal antibody (Clone CAM36, Kingfisher Biotech, Inc.), anti-bovine granulocyte (Clone CH138A, Kingfisher Biotech, Inc.), anti-bovine γδ TCR1-N24 δ chain (Clone GB21A, Kingfisher Biotech, Inc.), mouse anti-bovine CD4 (Clone ILA11A, Kingfisher Biotech, Inc.), mouse anti-bovine CD8α (Clone BAQ111A, Kingfisher Biotech, Inc.), and mouse anti-bovine CD21 (Clone GB25A, Kingfisher Biotech, Inc.) for 30 min at 4 ℃. Cells were incubated in complete RPMI (cRPMI) medium prepared with RPMI-1640 (Gibco, Carlsbad, CA) supplemented with 2 mM l-glutamine, 1% antibiotic antimycotic, 1 mM sodium pyruvate, and 10% autologous serum pooled from control-fed steers. Cells were washed twice with cRPMI and labeled with secondary cell surface antibodies: goat anti-mouse IgG1-AF647 (Southern Biotech), goat anti-mouse IgM-PE (Southern Biotech), goat anti-mouse IgG2b-PECy7 (Southern Biotech), anti-mouse IgG2a-PECy7 (BioLegend), goat anti-mouse IgM-AF647 (Southern Biotech), and goat anti-mouse IgG1-APCCy7 (Southern Biotech) at 4 ℃ for 30 min. Cells were washed twice with cRPMI, resuspended with flow cytometry staining (FACS) buffer, and analyzed on a Becton Dickinson FACSCantoII Flow Cytometry System. Data were analyzed by using FlowJo software 10.8.2 (FlowJo, LLC). A representative gating strategy for analyzed FluoZin-3 MFI in immune cell populations is included in Supplemental Fig. 1.

### Monocyte Purification

As previously reported, magnetic-activated cell sorting (MACS) isolation for CD14 + monocytes was performed on whole blood samples [27]. Monocytes were isolated by a positive selection using CD14 MicroBeads, human (Miltenyi Biotec, 130–097-052). Purified monocytes were resuspended in RNA*later* stabilization solution (Invitrogen, Life Technologies) in preparation for RNA isolation and stored at – 80 ℃.

### Granulocyte, NK Cell, B Cell, CD4, CD8, and γδ T Cell Purification

For fluorescent-activated cell sorting (FACS) isolation of remaining immune cells, cells from lysed whole blood were labeled with cell surface antibodies: anti-bovine granulocytes (Clone CH138A, Kingfisher Biotech, Inc.), anti-bovine γδ TCR1-N24 δ chain (Clone GB21A, Kingfisher Biotech, Inc.), mouse anti-bovine CD4 (Clone ILA11A, Kingfisher Biotech, Inc.), mouse anti-bovine CD8α (Clone BAQ111A, Kingfisher Biotech, Inc.), and mouse anti-bovine CD21 (Clone GB25A, Kingfisher Biotech, Inc.) at 4 ℃ for 30 min. Cells were then labeled with secondary cell surface antibodies: goat anti-mouse IgM-PE (Southern Biotech), goat anti-mouse IgG2b-PECy7 (Southern Biotech), anti-mouse IgG2a-PECy7 (BioLegend), goat anti-mouse IgM-AF647 (Southern Biotech), and goat anti-mouse IgG1-APCCy7 (Southern Biotech) at 4 ℃ for 30 min and resuspended with cRPMI for FACS sorting. Additional staining was performed with direct conjugate mouse anti-bovine CD335 IgG1-PE (Clone AKS1, Biorad) at 4 ℃ for 30 min. Immune cells were sorted using a BD FACSAria III Cell Sorting System (BD Bioscience, Ames, IA). Samples were sorted directly into RNA*later* stabilization solution (Invitrogen, Life Technologies) in preparation for RNA isolation and stored at – 80 ℃.

### RNA Isolation and cDNA Preparation

Purified immune cell populations were stored in RNA*later* stabilization solution (Invitrogen, Life Technologies) at – 80 ℃ until processing for quantitative real-time PCR (qPCR) analysis. According to the manufacturer’s instructions, RNA was isolated from sorted immune cell populations using MagMAXTM mirVana™ Total RNA Isolation Kit (Invitrogen, Life Technologies). The RNA concentration in each sample was determined using a Qubit™ RNA Broad Range (BR) Kit (Thermo Fisher Scientific) and Qubit™ 4 Fluorometer (Thermo Fisher Scientific). RNA (1000 ng per sorted sample) was DNase-treated, and cDNA was synthesized using random primers and Superscript III Reverse Transcriptase according to the manufacturer’s instructions (Invitrogen, Life Technologies).

### Quantitative Real-Time PCR Analysis

qPCR was performed using Power SYBR Green PCR Master Mix (Applied Biosystems, Carlsbad, CA), as previously reported [20]. Forward and reverse primers are compiled in Table 1. Primers for ZIP2, 4, 5, 8–14, ZnT2, 4, 6, 8, and MT1A and MT2A were designed using Integrated DNA Technologies (IDT) PrimerQuest™ Tool and validated in bovine liver samples. Expression for each gene was normalized to the housekeeping gene RPS9, from which ∆ cycle thresholds (∆Ct) were determined. Kara Thornton-Kurth from Utah State University kindly provided the primer sequences for ZIP7.

**Table 1:** List of sequences of primers used in the qPCR analysis to measure gene expression in sorted immune cells of steers. Blank spaces in the reference column indicate that the primers were designed using IDT PrimerQuest™ Tool and validated using bovine liver tissue

Gene	Accession #	Strand	Sequence (5′–3′)	Reference
SLC39A1	NM_00103581.2	Forward	TGCATGTGACGCTCCAGTTC	[28]
		Reverse	GTGGCCCACCATTCACTGTA	
SLC39A2	NC_037337.1:c26097864-26094678	Forward	GTGCTCTCCATCCTGTCTTTAG	
		Reverse	TCAGAGGGCGAAGTCATTTG	
SLC39A3	NM_001015521.1	Forward	CCCTCTGCCTAGCCCCATTA	[28]
		Reverse	GAAATCGATCTCGCCCTTGG	
SLC39A4	NC_037334.1:20936238–20946131	Forward	CTCTTGCTGCCCCTGGAC	
		Reverse	CCACCAGATCTGCGCGAG	
SLC39A5	NC_037332.1:c57097291-57091700	Forward	CTCTTTCACAGGCTTCTGCT	
		Reverse	CTAAATCCCGTGGCTCCTATTC	
SLC39A6	NC_037351.1:21006952–21028841	Forward	CCCTCCAAAGACCTATTC	[29]
		Reverse	ATCACCACTCAGTGTCCC	
SLC39A7	NC_037350.1:7386870–7391458	Forward	CATGCTCATGGTCACACACA	
		Reverse	TCCTCTGAGCTCTGTTTCTCC	
SLC39A8	NC_037333.1:22459057–22542658	Forward	GGAGTGGAGGGAAGAAAGAAG	
		Reverse	CTCACCTCGCCTGTGTATTT	
SLC39A9	NC_037337.1:81185412–81230624	Forward	CTGCTGGTGGTGATGCTAATA	
		Reverse	AGGGCTCACAAGTTCTCTTTC	
SLC39A10	NC_037329.1:84214336–84376926	Forward	CCCTGTTCTCCTGCCATAAAT	
		Reverse	GTGCCCAATGCTGAAAGTAATC	
SLC39A11	NC_037346.1:58126981–58503178	Forward	ATCACCATCCACAACATCCC	
		Reverse	ATCCCAATTCCAAGGGCTAAA	
SLC39A12	NC_037340.1:32374890–32459129	Forward	ACAGCTGCGAGGAGAACTACAGGCTCA	
		Reverse	GGTTTTGCATTTTCTGTTGGGGGTGTT	
SLC39A13	NC_037342.1:77240234–77248528	Forward	ACATCAAAGTCAGTGGCTATCTC	
		Reverse	GCTCTCTGACACACGCATATT	
SLC39A14	NC_037335.1:69680220–69731758	Forward	AGGCTCCTGCTCTACTTC	
		Reverse	AGCGTCTCAGAGGTATAATG	
SLC30A1	NM_001205893.2	Forward	GCAACTTGCTGGAAGCAGAA	[30]
		Reverse	TCAGGCTGAATGGTGGTAGC	
SLC30A2	NC_037329.1:127042849–127051974	Forward	GCCTATACTGTCTGTCCACATC	
		Reverse	CATGGCCTCCTTAAAGCATATTG	
SLC30A3	NC_037338.1:72369362–72382875	Forward	CATCAGCACCTTCCTCTTCTC	
		Reverse	CTCCCTGACACACACACTTT	
SLC30A4	NC_037337.1:64938017–64975659	Forward	ATCACCATCCACAACATCCC	
		Reverse	ATCCCAATTCCAAGGGCTAAA	
SLC30A5	NM_001192174.2	Forward	GGCTAAAATGGCTGAACACCC	[30]
		Reverse	ACACAAAGCCAGTACTAGCAACA	
SLC30A6	NC_037338.1:14763571–14812386	Forward	CCTAGCCCTGCCTATTCATTT	
		Reverse	AAGAGGAGGCATTGGGATTAC	
SLC30A7	NC_037330.1:c42410127-42310397	Forward	CAAGGTCCCAACATAAAC	[29]
		Reverse	AATGCAAAGAACTCCTCC	
SLC30A8	NC_037341.1:c47150546-47109476	Forward	GAGAATGAGGCCAGGTGATATT	
		Reverse	GATCGGGTCTGCCATCTTATAC	
SLC30A9	NC_037333.1:60859562–60948051	Forward	GCTTCGTAGGAGTGCTCG	[29]
		Reverse	GTGGGTTGCCTGTTATGG	
SLC30A10	NM_001192180.1	Forward	GCCCTGAATATCAGAGGGGT	[28]
		Reverse	GCTGGGGTCAATGTAGCACT	
MT-1A	NC_037345.1:c24025823-24023719	Forward	ATGGACCCGAACTGCTCCTGC	
		Reverse	GCGCAGCAGCTGCACTTGTCCG	
MT-2A	NC_037345.1:c24043331-24042365	Forward	ATCCTTTGCTCAGCAGTCTC	
		Reverse	ACAAACGGGTCAGGTTGTATTA	

### Statistical Analysis

**Fig. 2:**
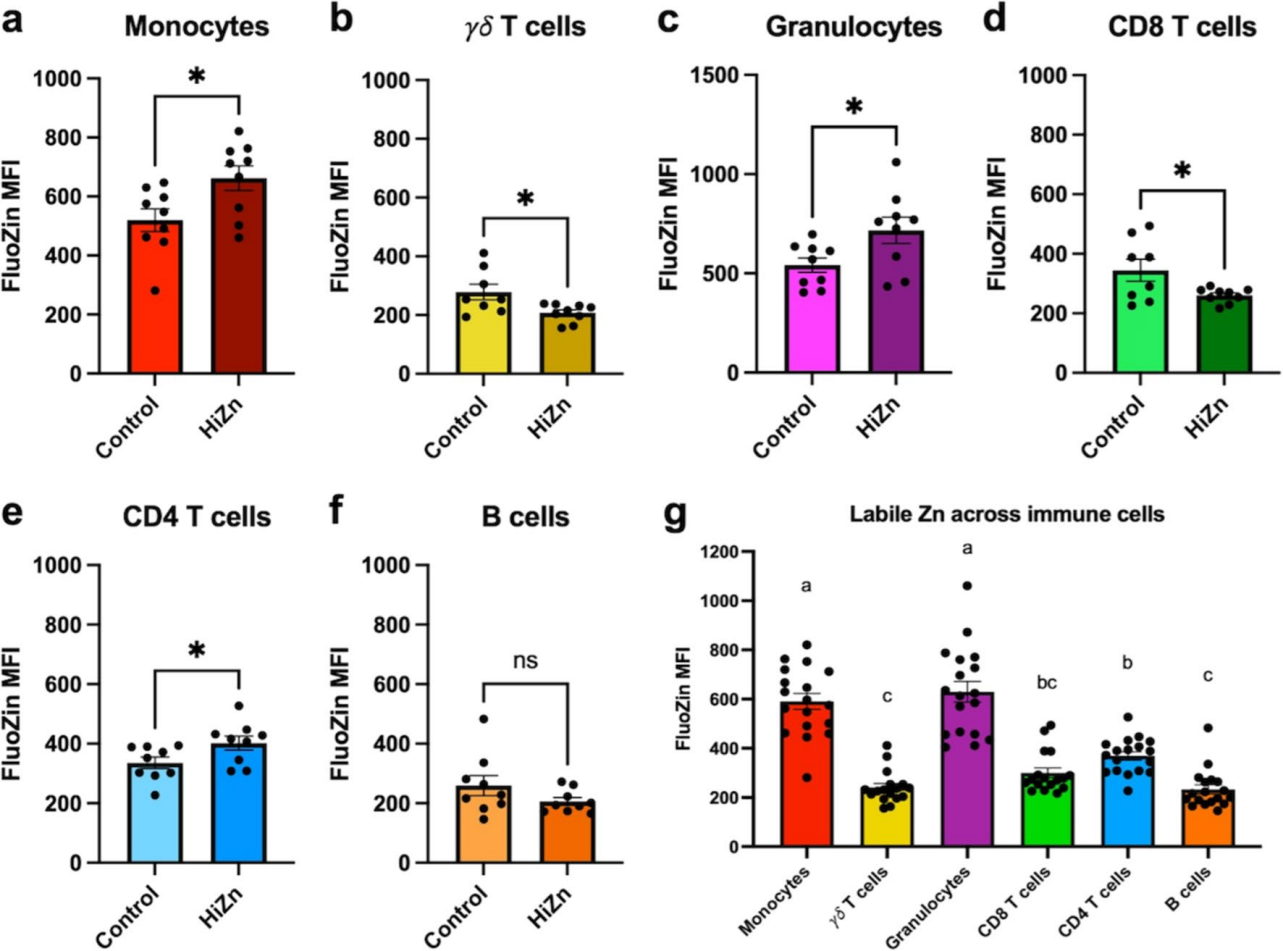
Labile Zn concentrations from circulating immune cells are influenced in steers fed the HiZn diet. Young, healthy-growing steers were fed a control diet with no Zn supplemented (*n* = 9) or a HiZn diet of 150 mg of Zn/kg DM (*n* = 9), as described in Rients et al. (manuscript in preparation). Peripheral blood was collected from control, and HiZn-fed steers on day 33 of dietary treatment. Whole blood was lysed, and 1 × 10^6^ cells were plated and incubated in FBS-free cRPMI for 30 min. Cells were loaded with FluoZin-3 and surface staining antibodies, then analyzed by flow cytometry to determine labile Zn concentrations based on FluoZin-3 MFI. The gating strategy is shown in Supplemental Fig. 1. Labile Zn concentrations estimated by FluoZin-3 MFI decreased in circulating **b** γδ^+^ T cells and **d** CD8^+^ T cells in steers fed a HiZn diet. In contrast, labile Zn concentrations accumulated in **a** CD14^+^ monocytes, **c** granulocytes, and **e** CD4^+^ T cells in steers fed a HiZn diet. **g** Bar graph of FluoZin-3 MFI, regardless of dietary treatment, in circulating immune cell populations (*P* < 0.05). Different letters show significant differences among cell types (*P* < 0.05). Data represent mean ± SEM. Data were analyzed using a standardized unpaired t-test

All statistical analyses were performed using Prism v9.5.1 (GraphPad Software, Inc.). The dietary treatment means ± the standard error of the means (SEM) were plotted for the FluoZin-3 and Zn transporter qPCR assay, and significant differences between treatment groups were determined using a standardized unpaired* t*-test. Outliers were detected using the ROUT method (Prism v9.5.1) and removed from data analyses when present. This included removal of 1 outlier from the monocyte Fluozin3 analysis (Fig. 2A) and 1 outlier from the CD8 T cell Fluozin3 analysis (Fig. 2D). The statistical analysis was performed using a sample size of 9 steers per dietary treatment, with the experimental unit being individual steers. Initial liver mineral concentrations were analyzed using pre-enrollment status as a covariate but this did not change the outcome and was not used for the analysis in the current manuscript. Plasma trace mineral was analyzed as repeated measures and included treatment × day interactions in the model. Additional details regarding liver and plasma trace mineral status and statistical analyses of dietary treatments are available in Rients et al. (manuscript under review).

## Results

### Liver and Plasma Zn Concentrations

Liver Zn decreased for both groups from days − 7 to 58 but did not differ between treatments (*P* > 0.05) at any timepoint (Table 2). Plasma Zn increased in both HiZn and control fed steers from day 0 to day 13. Plasma Zn was different between control and HiZn fed steers on d 28 (*P* = 0.006) but did not differ (*P* > 0.05) at any other timepoints (Table 2). Steers were within adequate ranges for plasma and liver Zn at all timepoints [13].

**Table 2:** Effects of dietary Zn supplementation on liver and plasma Zn concentrations of beef steers

Liver Zn mg/kg DM	Control	Zn150	SEM	*P* value
d -7	219	149	47.4	0.15
d 14	168	137	37.7	0.41
d 58	120	122	19.5	0.88
Plasma Zn mg/L	Control	Zn150	SEM	*P* value
d 0	1.05	0.97	0.089	0.41
d 13	1.15	1.15	0.095	0.94
d 28	1.06	1.33	0.086	0.006
d 57	1.14	1.28	0.092	0.17

^1^*Control*, no supplemental Zn; *HiZn*, 150 mg Zn/kg DM supplemented as ZnSO_4_

### Changes in Labile Zn Concentrations in Circulating Immune Cells After Dietary Zn Supplementation in Steers

To validate the FluoZin-3 assay, cells isolated from donor steers were labeled with FluoZin-3 dye and respective antibodies, and then left either untreated or stimulated with 50 µM of TPEN to create Zn-depleted cells or 100 µM of ZnPyr to create Zn-supplemented cells. Changes in labile Zn concentrations were then evaluated based on FluoZin-3 fluorescence, as shown in Fig. 1. To determine how dietary Zn supplementation influenced labile concentrations in circulating immune cells, peripheral blood was collected from control and HiZn-fed steers on day 33 of dietary treatment. Peripheral blood samples were loaded with FluoZin-3, stained with antibodies to cell surface antigens, and then analyzed by flow cytometry (Fig. 2). Results were gated on cell surface phenotype and analyzed for FluoZin-3 MFI as an indicator of intracellular labile Zn concentrations, as shown in Supplemental Fig. 1. Under normal physiological conditions, dietary Zn supplementation did not affect the frequency of circulating monocytes, granulocytes, γδ T cells, CD4 T cells, CD8 T cells, or B cells (Supplemental Fig. 2).

Circulating CD14^+^ monocytes, granulocytes (CH138A^+^), and CD4^+^ T cells from steers fed the HiZn diet had a higher FluoZin-3 MFI than control steers (*P* < 0.05, Fig. 2a, c, e). On the other hand, FluoZin-3 MFI was lower in γδ T cells (*P* = 0.02) and CD8α^+^ T cells (*P* = 0.03) from steers fed the HiZn diet than steers in the control diet (Fig. 2b, d**)**. Dietary Zn supplementation did not affect FluoZin-3 MFI in circulating CD21^+^ B cells (Fig. 2f). As shown in Fig. 2g, circulating monocytes and granulocytes had higher overall concentrations of labile Zn, measured by FluoZin-3 MFI, compared to CD4, CD8, γδ, or B cells (*P* < 0.0001). In addition, γδ T cells, CD8 T cells, and B cells had similar FluoZin-3 MFI levels, regardless of dietary effects (Fig. 2g). These results suggest dietary Zn supplementation modulates intracellular free Zn concentrations in circulating immune cells under normal physiological conditions.

### Changes in Gene Expression Levels of Zn Transporters in Sorted Innate and Adaptive Immune Cells from Steers Fed Supplemental Zn

**Fig. 3:**
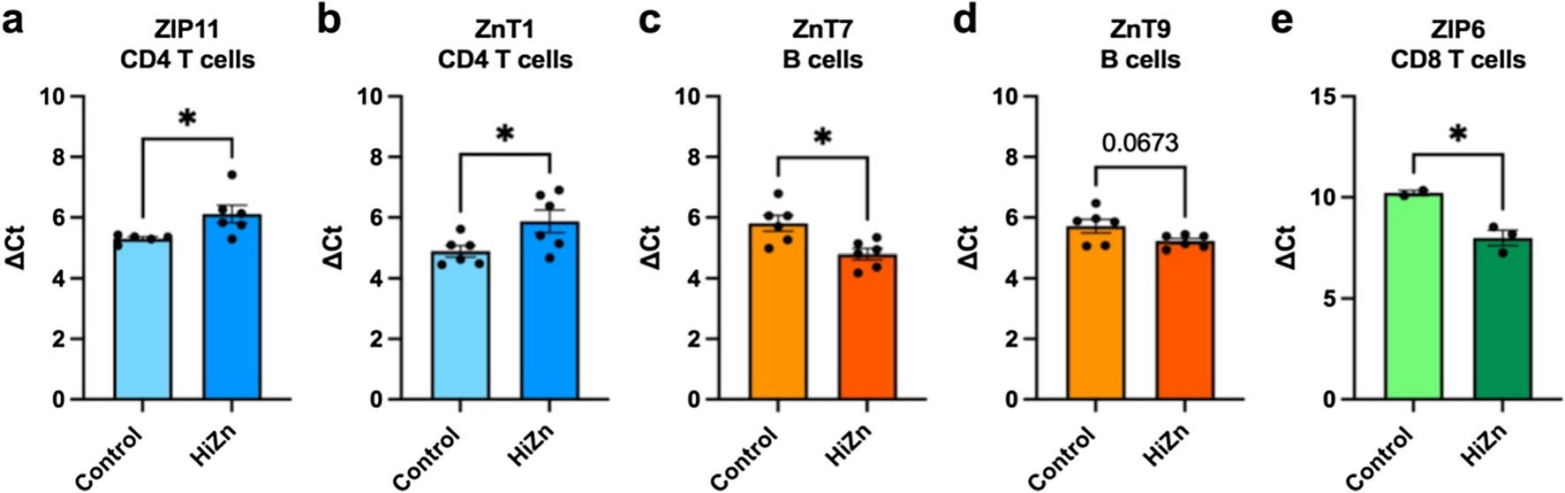
High dietary Zn supplements in steers modulated Zn transporter gene expression levels in circulating adaptive immune cells. On days 27 and 28, whole blood was collected from both dietary groups (control, *n* = 2–6, and HiZn, *n* = 3–6), and immune cell populations were sorted with MACS or FACS as described in materials and methods. Sorted cells were analyzed by qPCR for expressions of ZIP1–14, ZnT1–10, and MT1A and MT2A. For qPCR analysis, results were normalized to the housekeeping gene RPS-9, and gene expression values are shown as ∆Ct. A greater ∆Ct equals lesser gene expression. HiZn steers had lower mRNA expressions of **a** ZIP11 and **b** ZnT1 in sorted CD4^+^ T cells than control steers. In contrast, **c** ZnT7 increased, and **d** ZnT9 tended to upregulate in sorted CD21 + B cells from HiZn steers. **e** ZIP6 in sorted CD8^+^ T cells had higher mRNA expression levels from HiZn steers compared to control steers (*P < 0.05). Data represent means ± SEM. Data were analyzed using a standardized unpaired *t*-test

Since Zn transporters are crucial for modulating intracellular Zn concentrations, we next determined if dietary Zn supplementation modulated the expression of Zn transport machinery in circulating immune cells. Peripheral blood was collected from control and HiZn-fed steers on days 27 and 28 of dietary treatment. Circulating CD14^+^ monocytes, granulocytes, γδ TCR^+^ cells, CD335^+^ NK cells, CD4^+^ T cells, CD8α^+^ T cells, and CD21^+^ B cells were sorted, purified, and analyzed by qPCR for expression of ZIP1–14, ZnT1–9, and MT1A and MT2A (Fig. 3). ZIP11 and ZnT1 expression was downregulated (*P* < 0.05) in CD4 T cells from steers fed the HiZn diet compared to CD4 T cells from control steers (Fig. 3a, b). ZnT7 gene expression was higher (*P* = 0.01) in circulating B cells from steers fed HiZn, while ZnT9 tended to be higher than B cells from control steers (Fig. 3c, d). ZIP6 gene expression was upregulated (*P* = 0.02) in CD8 T cells from steers fed the HiZn diet; however, overall expression of ZIP6 was low in CD8 T cells, regardless of dietary treatment, and mRNA was only detectable in 2/6 controls and 3/6 HiZn samples (Fig. 3e).

### Gene Expression Profile of Zn Transporters in Circulating Sorted Bovine Immune Cells Under Normal Physiological Conditions

**Fig. 4:**
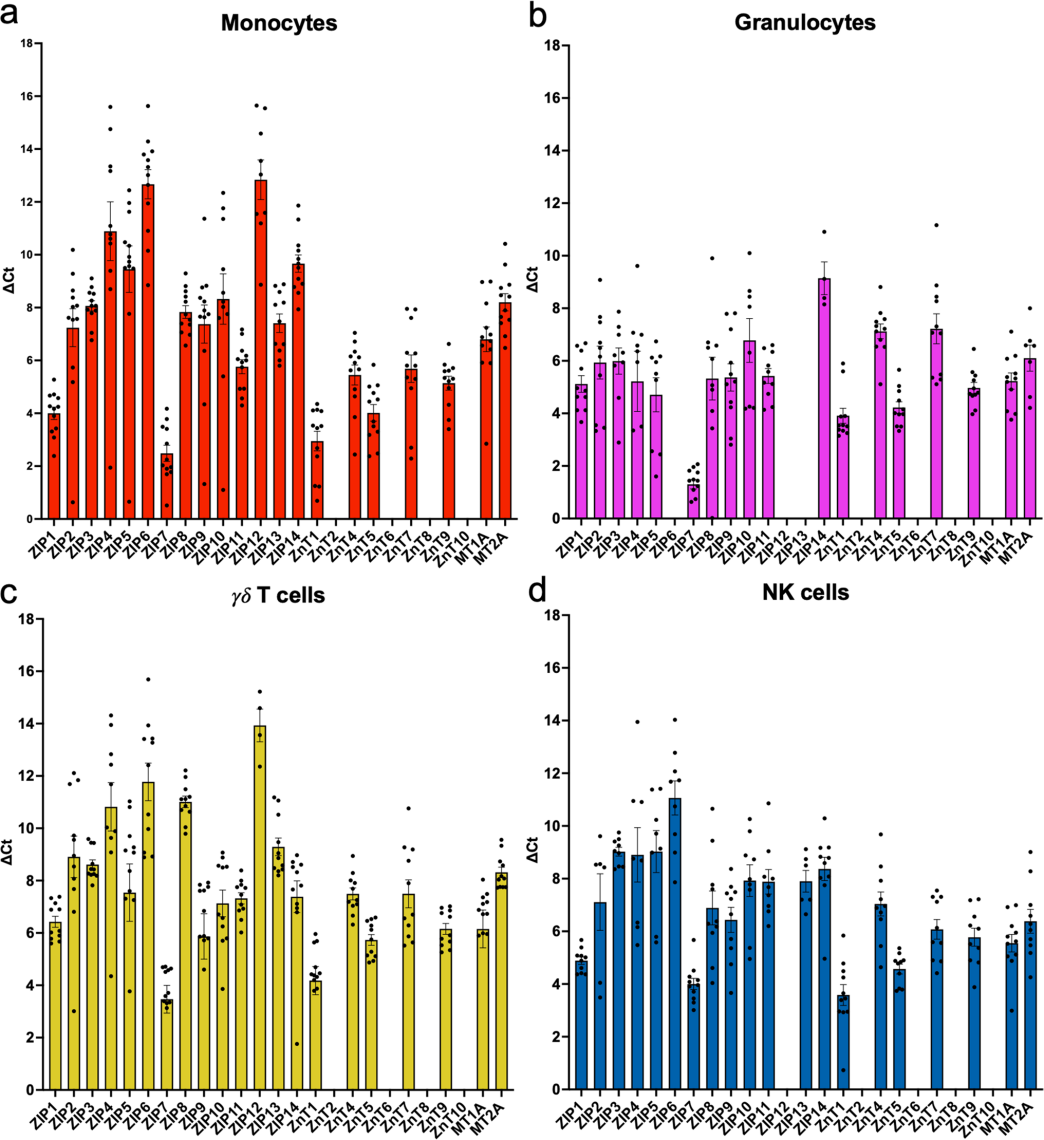
Bar graph of ZIP, ZnT, and MT gene expression levels in circulating innate immune cells. On days 27 and 28, circulating **a** CD14^+^ monocytes were isolated by MACS, **b** CH138A^+^ granulocytes, **c** GB21A^+^ γδ T cells, and **d** CD335^+^ NK cells were isolated by FACS from steers receiving no supplemental Zn diet (*n* = 6) or HiZn diet with 150 mg of Zn/kg DM (*n* = 6). MT and Zn transporter gene expressions were analyzed in sorted cells using RT-PCR. Data were normalized to the housekeeping gene RPS-9, and gene expression values are shown as ∆Ct. Data from both dietary groups were merged due to no statistical differences in treatment effects. Samples that did not amplify by qPCR were omitted; therefore, **a** *n* = 4–12; **b** *n* = 5–12; **c** *n* = 10–12; **d** *n* = 6–12. Missing bars represent genes that were not detected in sorted immune cell populations. Data represent mean ± SEM. Data were analyzed using a standardized unpaired *t*-test

**Fig. 5:**
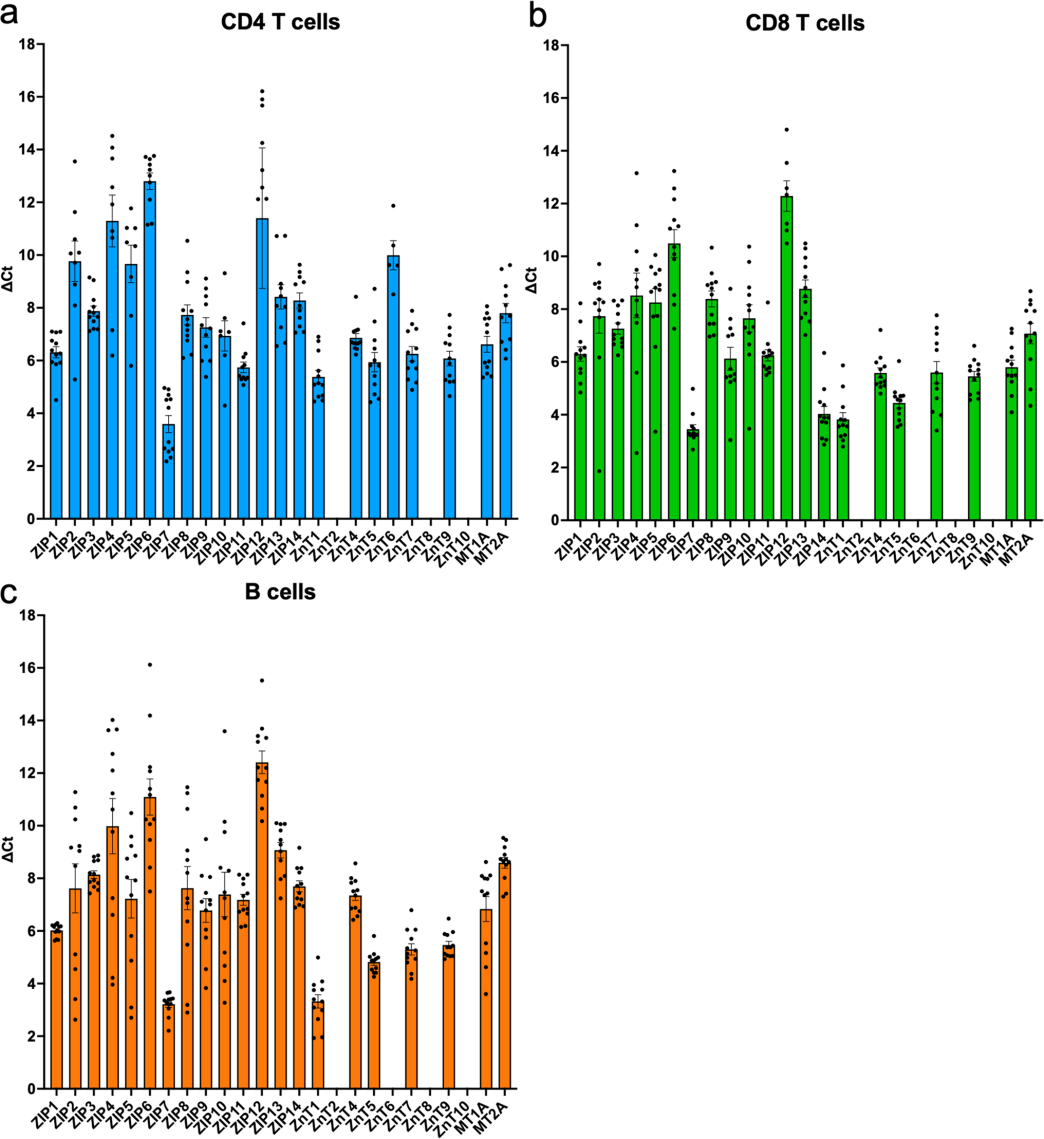
Bar graph of ZIP, ZnT, and MT gene expression levels in circulating adaptive immune cells. On days 27 and 28, circulating **a** CD4^+^, **b** CD8^+^ T cells, and **c** CD21^+^ B cells were isolated by FACS from steers receiving a no supplemental Zn diet (*n* = 6) or a HiZn diet, with 150 mg of Zn/kg DM (*n* = 6). MT and Zn transporter gene expressions were analyzed in sorted cells using RT-PCR. Data were normalized to the housekeeping gene RPS9, and gene expression values are shown as ∆Ct. Data from both dietary groups were merged due to no statistical differences in treatment effects. Samples that did not amplify by qPCR were omitted; therefore, **a** *n* = 5–12; (b) *n* = 5–12; (c) *n* = 5–12. Note that ZIP1 and ZnT1 in sorted CD4 T cells, ZIP12 in sorted CD8 T cells, and ZnT7 in sorted B cells had treatment effects, but data were merged in this graph for overall expression purposes. Missing bars represent genes that were not detected in sorted immune cell populations. Data represent mean ± SEM

Although it has been explored in rodents and humans, there has yet to be a comprehensive analysis of the Zn transport machinery in immune cells from ruminants. Therefore, we profiled the expression of all known bovine Zn transporters and MTs in purified immune cells from peripheral blood (Figs. 4 and 5). This analysis did not include ZnT3, MT3, and MT4 due to their known expression solely in brain cells and liver tissues, respectively [21, 22, 31]. Gene expression of several Zn transporters was measured in purified CD14^+^ monocytes, granulocytes (CH138A^+^), γδ TCR1^+^ T cells, and CD335^+^ NK cells (Fig. 4), CD4^+^ T cells, CD8α^+^ T cells, and CD21^+^ B cells (Fig. 5). Because we observed no differences in expression of these transporters between dietary treatment groups, the cell expression data from both control and HiZn-fed animals were combined for displaying data.

Of the 23 analyzed Zn transporters and two MTs, 19 Zn transporters and two MTs were ubiquitously expressed in monocytes from all 12 steers (Fig. 4a). ZIP1, ZIP7, and ZnT1 were expressed most highly in circulating monocytes. In granulocytes, 15 of 23 Zn transporters and two MT genes were expressed (Fig. 4b). ZIP7 was most highly expressed. ZIP6 and ZIP12, 13, and 14 were minimally expressed in granulocytes, and expression was undetectable in eight samples. Eighteen of the 23 Zn transporters and the two MTs were ubiquitously expressed in γδ T cells (Fig. 4c). ZIP7 and ZnT1 were the most expressed, whereas ZIP12 was minimally expressed and detected in only 6/12 samples. Sorted NK cell populations expressed 16/23 Zn transporters, and both MT genes were ubiquitously expressed in most samples (Fig. 4d). ZIP7 and ZnT1 were the most highly expressed, while ZIP5, ZIP6, and ZIP12 were minimally expressed or not detected in NK cells. ZnT2, ZnT6, ZnT8, and ZnT10 were not detected in any of the innate immune cells sorted. Missing gene expression bars on graphs indicate that the gene was not detected by qPCR.

From the 23 Zn transporters analyzed, 18 transporters and the MT genes were expressed in circulating CD4 T cells (Fig. 5a). ZIP6 was minimally expressed and detected in only 6/12 samples. ZIP12 was also minimally expressed in all samples. In circulating CD8 T cells, 18/23 Zn transporter genes and the MTs were detected (Fig. 5b). ZIP12 was minimally expressed and detected in only 10/12 samples. Lastly, 18/23 Zn transporters and both MTs were ubiquitously expressed in circulating B cells (Fig. 5c). Overall, ZIP7 was the most expressed gene of all Zn transporters, while ZnT2, ZnT6, ZnT8, and ZnT10 were minimally or not detected in any adaptive immune cells.

## Discussion

Plasma Zn concentrations are an inaccurate indicator of Zn status in cattle because they can respond to metabolic conditions unrelated to Zn status and are insensitive to marginal Zn fluctuations [15]. Labile Zn refers to a small fraction of the total Zn pool within cells that are readily exchangeable with extracellular Zn ions, meaning that it can quickly move in and out of the cell depending on the needs of the cell and the availability of Zn in the surrounding environment [3]. Labile Zn is involved in immunological functions such as cell signaling, proliferation, differentiation, and apoptosis [32–34]. FluoZin-3 is a permeable fluorescent dye that binds to free and loosely bound Zn ions [35] and measures intracellular Zn concentrations [35]. FluoZin-3 has been used to measure changes in Zn concentration over time [36] and in different cell compartments [37]. We postulated that FluoZin-3 labeling could predict changes in Zn availability and, therefore, be useful for assessing Zn status in different immune cells. The feedlot steers received a standard growing diet, which had around 58 mg Zn/kg DM, which is above NASEM’s 2016 recommendation, or supplemented with 150 mg Zn/kg DM (HiZn). We chose to supplement 150 mg Zn/kg in this trial because we have previously observed that feeding this concentration of Zn to finishing cattle increases cattle weight gain and meat quality at harvest [38]. We found that HiZn-fed steers had increased labile Zn in monocytes, granulocytes, and CD4 T cells from peripheral blood but decreased labile Zn in γδ and CD8 T cells (Fig. 2). Differences in FluoZin-3 fluorescence may indicate fluctuations in available Zn pools that impact immune cell signaling. Zn acting as signaling molecules can regulate the activity of major signaling molecules, including kinases, phosphatases, and transcription factors [39, 40]. However, since FluoZin-3 specifically binds to free and loosely bound Zn, the assay may not detect a portion of the available Zn in a cell that could be altered in response to dietary Zn intakes. We observed no change in labile Zn in circulating B cells on d 33 of dietary treatment. Although activation and proliferation by human B cells can elevate intracellular free Zn [41], there is no evidence that dietary Zn alters labile Zn concentrations in resting B cells. Moreover, although dietary Zn did not alter FluoZin-3 MFI, fluctuation of total Zn concentrations in B cells may have occurred. It may be interesting to measure total Zn content by ICP spectroscopy and with FluoZin-3 fluorescence to compare total and labile Zn concentrations as markers of Zn status.

Analysis of Zn transporter expression revealed several potential associations with Zn intake and status. Interestingly, despite a lack of change in free Zn, B cells from steers fed the HiZn diet upregulated the expression of ZnT7 and tended to upregulate the expression of ZnT9 (Fig. 3). ZnT7 is found in early secretory pathways, and its overexpression results in Zn accumulation in the Golgi of Chinese hamster ovary cells, whereas ZnT9 is ineffective at transporting Zn and instead works as a nuclear receptor coactivator [42, 43]. This suggests that ZnT7 and ZnT9 may not be involved in regulating free Zn in B cells but are influenced by dietary Zn intake. ZIP11 expression was downregulated in CD4 T cells from steers given the HiZn diet. ZIP11 is localized in the Golgi apparatus and is sensitive to dietary Zn intake, which might be a possible mechanism for lowering cytosolic Zn accumulation [44]. ZIP11 expression levels may negatively correlate with Zn availability in circulating CD4 T cells and serve as a potential biomarker for assessing Zn status. Additional research is necessary to establish whether Zn status assessment is consistent and reliable for measuring across various conditions.

Neither MT1A nor MT2A expression was affected in steers fed the HiZn diet. However, MTs have been implicated as possible indicators of human Zn status. Aydemir et al. [25] demonstrated that modest Zn supplementation in healthy young men increased the abundance of MT mRNA two-fold in leukocyte subsets and four-fold in dried blood spots within 6 days and that MT remained elevated at least 15 days following dietary treatment. In a study of middle-aged adults consuming a range of dietary Zn (9–31 mg/day), MT2A expression levels in PBMCs correlated with dietary Zn intake, while plasma Zn concentrations did not [24]. Another study reported increased MT mRNA in sort-purified human monocytes supplemented in vitro with Zn gluconate for 6 h [45]. Differences in methodology and treatment duration may contribute to differences in our observations or be due to species differences. Alternatively, the dietary Zn supplement offered by Aydemir et al*.* differed in Zn form and concentration, which might impact Zn transporter expression due to differences in bioavailability [25].

Previous human and mouse studies have described the expression profile of specific Zn transporters in different contexts, but similar data was unavailable for bovine species until this study. Wex et al*.* demonstrated nearly ubiquitous expression of ZIP1–14 and ZnT1–10 in human PBMCs, with all but ZIP5 and ZnT10 detected in at least some samples [46]. However, ZIP12 and ZnT3 were detected in only 1 sample, and ZIP2 was detected in only 3/5 samples. We did not detect ZnT10 in any sorted cell population, although we did observe reliable expression levels of ZIP2 in all cells analyzed. ZIP12 expression was inconsistent across bovine cell populations and was not detected in granulocytes or NK cells, suggesting this is only low or rarely expressed, much like in human PBMC. In contrast to Wex et al., which reported expression of ZnT2 and ZnT8 in human PBMC, we could not detect expression of either in any sorted population [46].

Overbeck et al. examined the expression of ZnT1–9 in cell lines, PBMCs, and primary T and B cells under conditions of Zn depletion or supplementation [23]. In our study, ZnT2 was not detected in B cells, T cells, monocytes, granulocytes, or NK cells. This agrees with Overbeck et al., who also failed to detect ZnT2 expression in primary human B and T cells [23]. In contrast, while expression of ZnT6 was observed in both primary human T and B cells, we did not detect ZnT6 expression in either population. Overbeck et al*.* stimulated primary human T cells with PHA and noted that ZnT6 expression was highly downregulated. We examined Zn transporter gene expression in circulating T cells under homeostatic conditions; therefore, ZnT6 expression may be modulated in bovine T-cell subsets during activation.

ZnT1 expression is the most dominant ZnT expressed in human PBMCs [23, 46] and functions to increase cytosolic Zn [47]. In our study of bovine cells, ZnT1 was also the most highly expressed across all the different immune populations (Figs. 4 and 5). Culturing human PBMCs in Zn concentrations ranging from 15 to 30 µM results in a threefold upregulation in ZnT1 [23]. In vivo, supplementing young men with 15 mg of Zn/day resulted in increased expression of ZnT1 in whole blood by day 2 of dietary treatment [25]. However, in our study, dietary Zn treatment in steers had the opposite effect on ZnT1 by decreasing its expression levels in circulating CD4 T cells. We examined ZnT1 expression in sorted immune cells rather than whole PBMC, which might be one possible explanation for this difference. In addition, the Aydemir et al. study used short-term dietary treatment, spanning 2 weeks [25], while our study examined ZnT expression after nearly 4 weeks of dietary Zn treatment at well above requirements. It is worth noting that ZIP7 was also a highly expressed transporter in both innate and adaptive immune cells in our study (as shown in Figs. 4 and 5). ZIP7 functions to import Zn from the endoplasmic reticulum to the cytoplasm, and a hypomorphic mutation in its gene is associated with immunodeficiency [48]. This transporter is crucial in B cell development, and in mice with ZIP7 deficiency, cytoplasmic Zn accumulation is observed, leading to increased phosphatase activity and decreased phosphorylation of signaling molecules downstream of the pre-B and B-cell receptors [48]. However, our results indicated dietary Zn supplementation in steers did not affect labile Zn concentration or the expression of ZIP7 in B cells, suggesting Zn homeostasis is stable in resting B cells. More research is needed to determine whether labile Zn concentration or gene expression of Zn transporters changes in activated immune cells.

In conclusion, we have shown dietary Zn supplementation to growing steers changes labile Zn concentration and the expression of Zn transporter genes in immune cells in the bloodstream. This study also provides a comprehensive Zn transporter gene expression profile in circulating bovine immune cells. Further research will continue to validate the usefulness of Zn transporters and labile Zn concentrations as biomarkers of Zn status in cattle under conditions of homeostasis, stress, or infection.

## Supplementary Information

Below is the link to the electronic supplementary material.

Supplementary file1 (DOCX 551 KB)

## Data Availability

The raw data supporting the conclusions of this article will be made available by the authors without undue reservation.
