# Supplementary material for: Dietary Zinc Supplementation in Steers Modulates Labile Zinc Concentration and Zinc Transporter Gene Expression in Circulating Immune Cells
Source: Biol Trace Elem Res. 2024 Mar 5;202(12):5489–501. doi: 10.1007/s12011-024-04123-6 (PMC11502596; doi:10.1007/s12011-024-04123-6)
Supplement: Supplementary file 1 — Supplementary file1 (DOCX 551 KB) [file 12011_2024_4123_MOESM1_ESM.docx]

# Dietary zinc supplementation in steers modulates labile zinc concentration and zinc transporter gene expression in circulating immune cells

Carlos E Franco^1^, Emma L Rients^2^, Fabian E Diaz^1^, Stephanie L Hansen^2^, Jodi L McGill^1*^

^1^Department of Veterinary Microbiology and Preventive Medicine; Iowa State University, Ames, IA, USA

^2^Department of Animal Science, Iowa State University, Ames, IA, USA

##

* **Corresponding author:** Jodi L McGill, Department of Veterinary Microbiology and Preventive Medicine, Iowa State University, 1907 ISU C-Drive, Ames, IA, United States. Email address: jlmcgill@iastate.edu


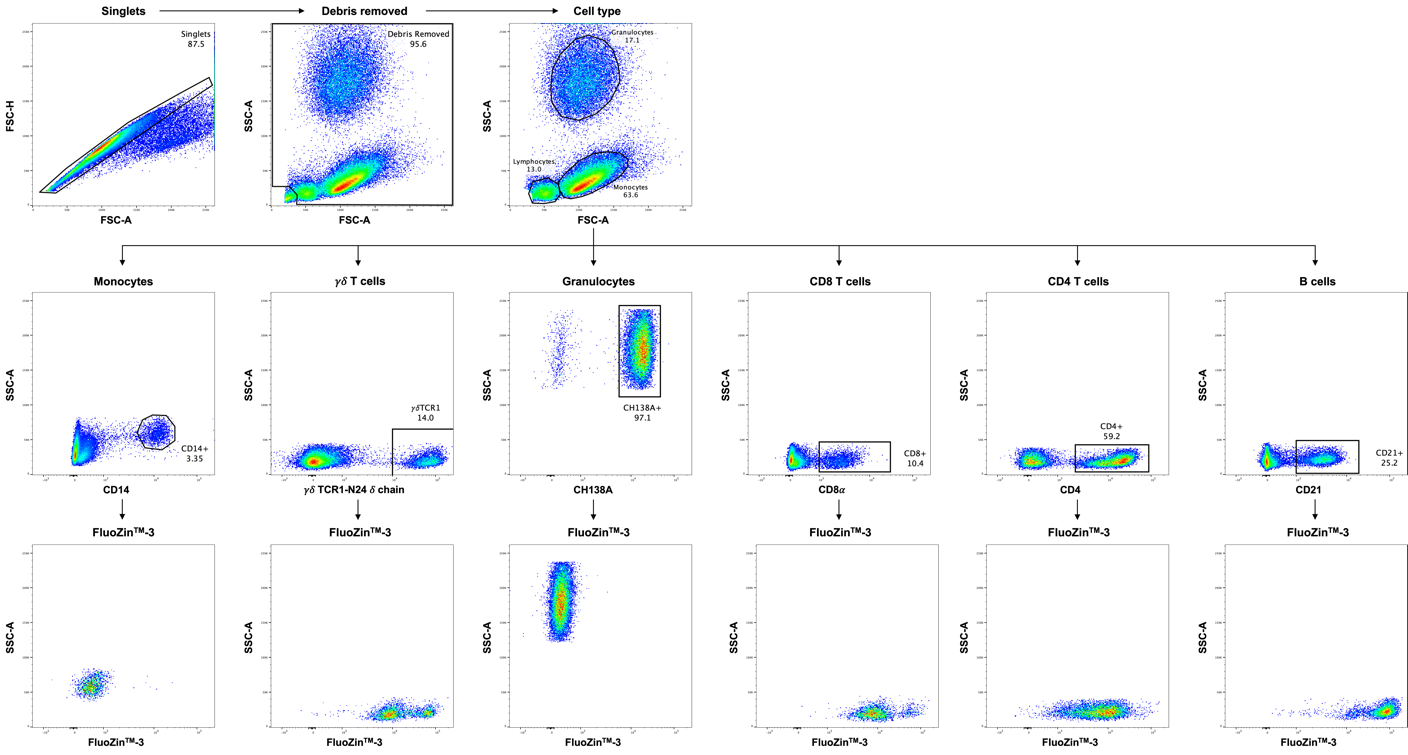


**Supplemental Figure 1.** **Gating strategy by flow cytometry for innate and adaptive circulating immune cells from steers fed dietary Zn supplements.** The figure depicts the gating strategies used to identify monocytes, 𝛾𝛿 T cells, granulocytes, CD8 T cells, CD4 T cells, and B cells present in whole blood of young, healthy growing Angus crossbred steers. After removing doublets and

debris, immune cell types, such as granulocytes, lymphocytes, and monocyte, were selected based on forward and side scatter. Granulocytes (CH138A+) were identified from the granulocyte forward and side scatter gate, and FluoZin-3 mean fluorescence intensity (MFI) was measured from the entire population, as gating for FluoZin-3 would exclude cells with low FluoZin-3. Monocyte subsets (CD14+), B cells (CD21+), and different T cell subsets (CD4+, CD8𝛼+, and 𝛾𝛿) were also identified using the same gating method based on forward and side scatters. Data represent mean ± SEM


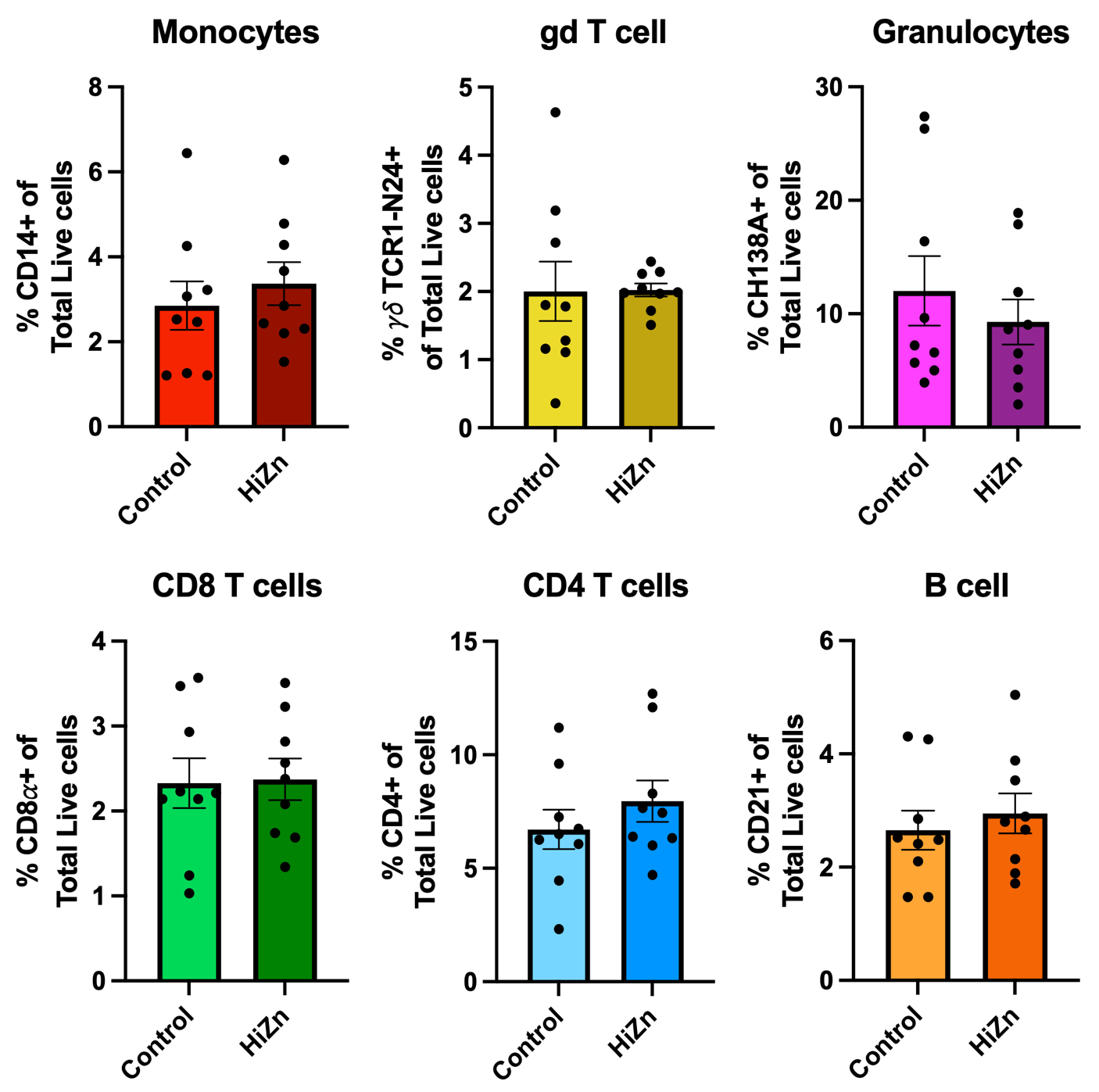


**Supplemental Figure 2.** Frequency of circulating immune cells does not change in Zn-supplemented steers. Whole blood cells were loaded with FluoZin-3 dye and stained with cell surface antibodies to determine labile Zn concentrations in different immune cell populations. Samples were analyzed by flow cytometry and were analyzed for the frequency of each circulating population from total live cells using the gating strategy shown in Supplemental Figure 1. Data represent mean ± SEM and were analyzed using a standardized unpaired t-test.
